# Learner satisfaction-based research on the application of artificial intelligence science popularization kits

**DOI:** 10.3389/fpsyg.2022.901191

**Published:** 2022-07-19

**Authors:** Yingfei Ling, Zhou Jin, Yingxin Li, Jieya Huang

**Affiliations:** ^1^College of Science and Technology, Ningbo University, Cixi, China; ^2^College of Educational Science and Technology, Zhejiang University of Technology, Hangzhou, China

**Keywords:** maker education, learners’ satisfaction, influencing factors, artificial intelligence, science kit

## Abstract

The application of artificial intelligence science popularization kits in maker courses has promoted the rapid development of maker education. However, there exist few theoretical and empirical studies on the application of artificial intelligence science popularization kits in maker education. The theory of learner satisfaction can be used to explain learner motivation and outcomes with regard to participation in maker education using the artificial intelligence suite. Therefore, taking advantage of the opportunity the Zhejiang Action Plan for Promoting the Development of New Generation Artificial Intelligence (2019–2022) has provided, this study first conducted semi-structured interviews based on the results of a literature review and a questionnaire survey and then performed Pearson correlation analysis and regression analysis using SPSS 24.0 to explore the influencing factors of students’ satisfaction with the use of artificial intelligence science popularization kits in education. The following results were obtained. (1) The correlation between grades and learners’ satisfaction is not significant. (2) The use of a high-quality artificial intelligence science suite in the classroom will positively impact learners’ satisfaction. (3) The degree of interaction with the artificial intelligence suite is negatively correlated with learners’ satisfaction. (4) Teaching adaptability is significantly positively correlated with learner satisfaction. (5) Learners’ individual characteristics have no significant positive correlation with learner satisfaction. Therefore, this study recommends focusing on suite quality, improving human–computer interaction, adopting a student-centered strategy, and aiming at improving the suitability of the curriculum.

## Introduction

The 2021 Zhejiang Artificial Intelligence Industry Development Report points out that the market scale and market benefit of the Zhejiang artificial intelligence (AI) industry have improved significantly, and several industry-leading AI enterprises and sub-industries have been formed. In addition, using the AI suite to teach AI courses aimed at cultivating AI talent provides technical support for promoting the reform that seeks to combine teaching theory with practice, enhance the learning experience, and improve learning quality. Therefore, schools and teachers need to effectively improve students’ satisfaction with using AI kits in maker education, so that the students are willing to use the AI suite during maker education, which will improve their comprehensive abilities such as the practical and thinking abilities (Liu, 2000; Yang and Zhao, 2018).

To date, some researchers have discussed the application of the AI science popularization suite. Kafai et al. (2014) introduced electronic textiles to the American–Indian seventh and eighth graders to assist in the teaching of engineering and computing. Researchers pointed out that accenting students’ interests with technology will usher students into the digital age. Galadima (2014) introduced the Arduino microcontroller to learning as a primary means of stimulating students’ understanding of the electronics and programming. Moreover, according to Brown (2015), complex tools (such as 3D printing software and 3D printers) are often used in production, which enables students to participate in hands-on learning and problem solving. By analyzing the curriculum, Kim (2017) explored primary school content suitable to be taught with 3D printers to meet students’ developmental needs and improve their satisfaction with the curriculum. Therefore, this study deemed it necessary to explore the influencing factors of students’ satisfaction with the AI science popularization suite in maker education through a questionnaire survey and theoretical reasoning.

According to a review of the literature on the AI science popularization kit, the AI kit comprises various resources used to deliver AI education through teaching activities (Yin, 2011). The kit covers the configuration of equipment (Zhao and Hu, 2005) including but not limited to digital electronic equipment such as open source hardware, design, and manufacturing equipment such as a laser cutting machine, and 3D printers and mechanical processing (Wu and Zhong, 2018). However, at present, there exist few theoretical and empirical studies on students’ satisfaction with the AI science popularization suite in maker education. This study answers the following questions:

1. What methods should teachers and schools adopt to improve students’ satisfaction with using the AI suite?
2. Does learner satisfaction differ according to AI suite quality and/or because AI kits vary in terms of the degree of interactivity?
3. Does learner satisfaction differ according to the adaptability of both the AI suite and the curriculum?
4. Does learner satisfaction differ according to learners’ individual characteristics?

## Literature review

### Research status of maker education

AI is an interdisciplinary subject that simulates human abilities and intelligent behavior (Luckin et al., 2016). Zhong (2017) asserted that AI advancements will drive innovation in and the development of human science and technology, the economy, society, culture, the military, and other fields. Yan et al. (2017) noted that the influence of AI technology on human society is becoming increasingly profound and extensive and that it is providing many new development opportunities in agriculture, medical treatment, education, energy, national defense, and several other fields. Yu (2018) offered the view that AI is changing our social production and lifestyles. Given the rapid development that characterizes our times, AI technology’s influence on all aspects of society is indeed becoming increasingly profound, and the field of education is an important AI application area (Duncan-Howell, 2010).

The maker movement is theoretically rooted in Piaget (1964); Dewey (1974), and Montessori (1976); (Martinez and Stager, 2013). With the maker movement sweeping the world, maker education has garnered much attention, and countries all over the globe have implemented educational reforms in keeping with the times. Maker education is a means of attracting students to science, technology, engineering, and mathematics (STEM) courses and cultivating their creative thinking. Maker teaching usually employs educational aids such as 3D printing software and 3D printers. Amidst the Internet Plus era, the integration of maker education and information technology has gradually deepened. Representative of emerging technologies, the application of the AI science popularization suite to teaching has become one of the research focuses of the maker education.

The maker education is one of the manifestations of the maker movement, which is strengthening in the education sector. It refers to a new education model that focuses on practice, creation, and learning, and trains innovative talent (Han and Ding, 2017). Based on the educational motto “learning by doing” (Gentry, 1990), maker education is for students in all grades (Stager, 2014). It is implemented *via* the project-based teaching method or the flipped classroom (Helle et al., 2006; Franklin et al., 2013).

Maker education differs from general education in that it promotes a teaching concept shift. Compared with general education, which is delivered through indirect knowledge, maker education emphasizes “teaching in practice” (Martinez and Stager, 2013). In addition, compared with the teacher-centered general education, maker education is student-centered (Buskirk, 1976); that is, students are encouraged to participate actively in learning, complete specific tasks, and solve problems through mutual cooperation (Donnelly and Fitzmaurice, 2005), and build new knowledge based on existing knowledge (Young and Collin, 2004). Furthermore, while in general education, teachers emphasize learning outcomes, maker education promotes a change in teachers’ role (Rosen and Weil, 1995); that is, teachers’ pay more attention to the learning process (Wurdinger and Carlson, 2009).

### Learning satisfaction theory

Satisfaction refers to a person’s emotional state after realizing their expected performance (or outcome) (Taylor, 1996). The concept of satisfaction was first introduced in the field of economics, resulting in the famous customer satisfaction theory. For consumers, satisfaction is premised upon their satisfaction reaction, which is a judgment of their pleasure level arising from the consumption of products and services (Dietz, 1997). Affected by quality expectations, consumers’ satisfaction is usually linked to product performance expectations based on their previous impressions of the particular product or service (Solomon, 1996). The definition of customer satisfaction focuses on expectations, experience, perceived service quality, and the resulting evaluation and has been applied to many professional services, such as medical care and various governmental and non-profit organizations’ activities. Marketing researchers have analyzed the theory of customer satisfaction in the context of higher education (Taylor, 1996). Füller and Matzler (2008) empirically investigated different factors’ roles in different market segments. Aziri (2011) examined the influence of job satisfaction on enterprise management and found that job satisfaction has a strong influence on employees’ work motivation and indirectly affects commercial organizations’ performance. Ross et al. (1995) used several measurement standards, including the visual simulation scale on a Likert 5-point scale, to evaluate patients’ satisfaction (Gould et al., 2001; Allen and Seaman, 2007).

Studies have shown that, compared with traditional classrooms and fully virtual teaching, classrooms that use the AI science popularization suite can improve learners’ satisfaction to a certain extent. To this end, Li and Zheng (2016) have recommended that the maker classroom first ensure the selection of an appropriate AI suite. Furthermore, Liu and Wang (2019) examined the influencing factors of learners’ satisfaction from an interaction perspective and found that the degree of interactivity positively impacts classroom satisfaction. In foreign countries, improved student satisfaction is related to the enhancement of students’ abilities, and the use of kits significantly impacts students’ computational thinking, logical analysis, and comprehensive application abilities. Kit application should prioritize meeting students’ developmental needs. Compared with the traditional classroom, learners’ enhanced abilities and acceptance of the suite can promote high-learner satisfaction levels. Learners’ satisfaction indicates their inner recognition of the use of AI science teaching aids. It is therefore important to devise an effective strategy for building a teaching system that utilizes AI science teaching aids and provides targeted services for learners to improve their satisfaction and ultimately support the success of AI teaching.

Satisfaction refers to learners’ positive (i.e., happiness) feelings about or attitude toward learning activities (Long, 1985). When the users’ satisfaction with a certain course is low, they will tend to resist and reject it and may express unwillingness to continue enrolment (DeLone and McLean, 2003). According to Knowles (1970), satisfaction refers to learners’ pleasant feelings or attitude toward learning activities. Therefore, the theory of learning satisfaction can be used to explain the motivation for and results of learners’ participation in maker education.

### Research status of satisfaction with the artificial intelligence science suite in maker education

#### Kit quality

Experimental inquiry is at the core of maker education (Olympiou and Zacharia, 2018), and it cannot be done without experimental tools. High-quality teaching aids are a prerequisite for improving students’ satisfaction. Teaching aids used in science classes should be easily accessible and of high quality (Bouck and Flanagan, 2010). The quality problem with “equipment allocation in maker space” has become one of the research focuses of maker education (Krueger, 2014). Operating high-quality kits can induce more task-related cognitive behaviors (Antle, 2013) and improve students’ logical thinking and practical abilities. Based on the earlier discussion, this study proposes hypothesis 1:

H1: The quality of the artificial intelligence suite is positively related to learner satisfaction.

#### The suite’s degree of interactivity

In teaching, the three basic interactions are student–teacher interaction, student–student interaction, and student–content interaction (Moore, 1989). In this context, the AI suite is an interactive medium. Relevant research has shown that teaching aids with interactive features can help students effectively engage in cognition, thus, improving their learning effectiveness (Plass et al., 2009), and embed learners’ cognitive activities into the environment, thereby reducing cognitive load (Antle, 2013). Teaching aids with rich interactive functions can also promote students’ perception of the controllability of teaching aids and guide learners to take relevant corresponding actions (Pouw et al., 2014). Based on the aforementioned discussion, this study proposes hypothesis 2:

H2: The artificial intelligence suite’s degree of interactivity is positively related to learner satisfaction.

#### Teaching adaptability

At present, some iterations of maker education do not follow the established rules and lack a scientific design and the integration of basic subject knowledge (Yu and Hu, 2015). Such versions of maker education cannot effectively meet students’ learning needs and do not have teaching adaptability. Wu and Zhong (2018) asserted that the teaching adaptability of maker educational equipment should be evaluated from two aspects, namely, product quality and teaching nature, to promote the important role of teaching equipment in maker classrooms (Liu et al., 2017). Based on the aforementioned discussion, this study proposes hypothesis 3:

H3: Teaching adaptability is positively related to learner satisfaction.

#### Individual learner characteristics

In the maker education, providing students with personalized and adaptive services is an important goal. Studies have shown that learners may only adopt active learning strategies if they identity with both the learning media and the learning environment (Liu and Jiang, 2004). Learners with a strong learning ability more positively perceive their learning achievements and teaching satisfaction. It is therefore necessary to monitor differences and changes in learners’ individual characteristics and promptly adjust the application of teaching kits to improve learner satisfaction (Peng and Zhu, 2019). Based on the above discussion, this study proposes hypothesis 4:

H4: Individual learner characteristics are positively related to learner satisfaction.

**FIGURE 1:**
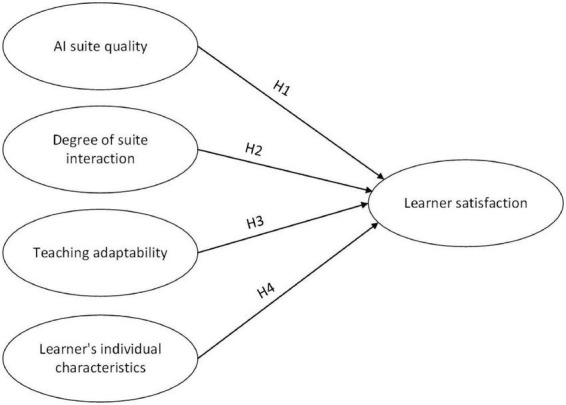
Research hypothesis.

Therefore, the proposed research structure diagram is shown in Figure 1.

## Methodology and materials

### Questionnaire design

This study is based on consumer satisfaction theory, with reference to relevant extant literature. The main factors that affect students’ satisfaction with AI science popularization suite courses are suite quality, degree of interactivity, teaching adaptability, and individual learner characteristics. In the questionnaire, the influencing factors of satisfaction, with four secondary attributes, are reflected in 36 questions: nine on suite quality, seven on degree of interactivity, ten on teaching adaptability, and ten on individual learner characteristics. All the items were measured on a 7-point Likert scale, ranging from 1 = *totally disagree* to 7 = *totally agree* (Allen and Seaman, 2007).

### Research object

To further verify the influence of suite quality, degree of interactivity, teaching adaptability, and individual learner characteristics on satisfaction with the AI science popularization suite, this study, by virtue of the comprehensive Zhejiang Action Plan for Promoting the Development of New Generation Artificial Intelligence (2019–2022), conducted a questionnaire survey in colleges and universities in 2021, targeting students who participated in an AI science popularization suite course and possessed a certain level of knowledge and logical analysis ability.

### Semi-structured interviews

After designing the first draft of the questionnaire, this study analyzed the content of the semi-structured interview. Before finalizing the questionnaire, 32 graduate and undergraduate students from Z University who participated in a course using the AI science popularization suite were randomly selected and interviewed to ascertain their self-reported satisfaction with the suite. Interview questions included frequency of contact with courses using the AI suite, whether the AI suite can meet actual classroom needs, and consequences of using the AI suite.

The statistical results are shown in Table 1. Analysis of the semi-structured interview transcripts confirmed that the influencing factors of undergraduates’ satisfaction with AI science popularization suite courses include suite quality, degree of interactivity, teaching adaptability, and individual learner characteristics. Based on the results of the semi-structured interviews, two university professors were invited to revise the first draft of the questionnaire. This entailed deleting repeated questions and modifying unclear questions. The final questionnaire contains 16 questions, none of which are reverse scoring questions to ensure the effectiveness and utility of this research.

**TABLE 1:** Students’ (dis)satisfaction with extracurricular activities.

Satisfactory aspects of the AI science popularization kit course	Frequency	Satisfactory aspects of the AI science popularization kit course	Frequency
Task completion	25	High quality	18
High sense of accomplishment	8	High degree of intelligence	12
High degree of interactivity	3	Compatible with the course objectives	5
Good learning atmosphere	6	Elicits a range of emotions	9
Aids knowledge comprehension	4	Rigorous kit operation	10
Contributes to enhanced abilities	4	Less activity time	6
Promotes group cohesion	3	Adequately serves multiple functions	3

### Implementation of research tools

A total of 132 questionnaires were collected through the Questionnaire Star System (WJX) system as shown in Table 2. In total, twelve invalid questionnaires that were completed in less than 30 s or were traced to duplicate login accounts were deleted, leaving 120 valid questionnaires for analysis. Among these, 105 respondents were undergraduates, who accounted for 79.5% of the total number of the respondents. The remaining 27 respondents were graduate students, who accounted for 20.5% of the total sample.

**TABLE 2:** Respondents’ demographic characteristics.

Category	Attribute	Quantity	Proportion
Gender	Male	73	60.8%
	Female	47	39.2%
Level of study	Freshman	22	18.3%
	Sophomore	20	16.7%
	Junior	38	31.7%
	Senior	13	10.8%
	Grade 1 master	5	3.3%
	Grade 2 master	16	13.3%
	Grade 3 master	6	5.0%
Frequency of exposure to courses using the AI suite			
	Never	7	5.9%
	Rarely	12	10%
	Sometimes	28	23.3%
	Often	44	36.7%
	Always	29	24.1%

## Results and discussion

### Reliability and validity tests

Before administering the questionnaire, this study conducted a pilot test to improve the questionnaire’s content validity by identifying and eliminating ambiguity in the content, specifically in the phrasing of the questions (Churchill, 1979). The pilot test involved 54 college students from two classes. According to Hair et al. (2006), when Cronbach’s alpha coefficient is greater than 0.7, the questionnaire indicators have good reliability. Therefore, in this study, Cronbach’s alpha for internal consistency was used to analyze the indicators’ reliability. The results show that the Cronbach’s alpha for all the indicators is 0.945, which exceeds the 0.7 threshold; moreover, the Kaiser–Meyer–Olkin value is 0.874, indicating that the questionnaire has good reliability and validity.

In this study, all the survey data were input into SPSS 24.0, and the influence of various related factors on learners’ satisfaction was studied using the Pearson’s correlation analysis and regression analysis.

### Pearson’s correlation analysis

Pearson’s correlation coefficient is essentially a linear correlation coefficient among the statistical methods, and its analysis is usually used to measure the linear relationship between fixed-distance variables (Zhang et al., 2014). According to the survey data, 88.17% of undergraduates and 81% of postgraduates were satisfied with the use of the AI science popularization suite in their courses. *Via* Pearson’s correlation analysis, this study obtained results for learner satisfaction, as shown in Table 3. The Pearson’s correlation coefficients for suite quality, curriculum suitability, individual improvement of students’ abilities, human–computer interaction, and learners’ satisfaction all exceed 0, with a *P*-value of 0.000 (*P* < 0.05). Hence, a positive correlation was found.

**TABLE 3:** Correlation analysis of learner satisfaction with suite quality, curriculum suitability, improvement in individual students’ abilities, and human–computer interaction.

		Kit quality	Curriculum suitability	Individual improvement	Human–computer interaction
Degree of satisfaction	Pearson’s correlation	0.692**	0.778**	0.876**	0.787**
	Sig.	0.000	0.000	0.000	0.000
	Number of cases	120	120	120	120

**At the 0.01 level (double tail), the correlation is significant.

In this study, Pearson’s correlation analysis was used to assess the relationship between learners’ grades and learner satisfaction, as shown in Table 4. The Pearson’s correlation coefficient between undergraduate and graduate students’ grades and learner satisfaction is greater than 0, while the Sig value exceeds 0.05, and the *P*-values are 0.574 and 0.496 (*P* > 0.05), indicating an insignificant correlation.

**TABLE 4:** Correlation analysis of learner satisfaction and learners’ grades.

		Undergraduates’ grades	Graduates’ grades
Degree of satisfaction	Pearson’s correlation	0.059**	0.137**
	Sig.	0.574	0.496
	Number of cases	93	27

**At the 0.01 level (double tail), the correlation is significant.

As shown in Table 5, this study performed Pearson’s correlation analysis of learners’ participation in AI suite courses and learner satisfaction. Table 3 shows that the Pearson’s correlation coefficient between the frequency of learners’ exposure to courses using the AI suite and students’ satisfaction is greater than 0, and the *P*-value is 0.524 (*P* > 0.05), indicating that there is no correlation.

**TABLE 5:** Correlation analysis of learner satisfaction and frequency of exposure to courses using the AI suite.

		AI suite exposure frequency
Degree of satisfaction	Pearson’s correlation	0.524**
	Sig.	0.005
	Number of cases	27

**At the 0.01 level (double tail), the correlation is significant.

### Regression model analysis

Multiple regression analysis is a statistical analysis method (Wang et al., 2008) to determine whether there is a linear or non-linear relationship between multiple independent variables and a single dependent variable. The main objectives are to analyze the quantitative relationship between several independent variables and a single dependent variable, explain the influence of the independent variables on the dependent variable, and potentially make predictions. Therefore, this study adopted a multiple regression analysis model to analyze the influential relationship between dependent and independent variables. According to the survey data, suite quality, curriculum suitability, improvements in individual students’ abilities, and human–computer interaction are positively related to learner satisfaction. The results of regression model analysis of learner satisfaction are shown in Table 6. The coefficients of relevance, interactivity, and learner satisfaction all exceed 0.05, but none are significant. The values for quality, improvement in individual students’ abilities, and learner satisfaction are less than 0.05, indicating significance.

**TABLE 6:** Retrospective analysis of learner satisfaction and quality, suitability, improvement in individual students’ abilities, and human–computer interaction.

Model	Non-standardized coefficient		Standardized coefficient	t	Significance
	B	Standard error	Beta		
(Constant)	0.084	0.676		0.124	0.903
Quality	0.258	0.083	0.376	3.124	0.005
Suitability	–0.063	0.085	−0.102	−0.742	0.466
Individual improvement	0.423	0.067	0.705	6.322	0.000
Interactivity	0.055	0.084	0.070	0.655	0.519

**FIGURE 2:**
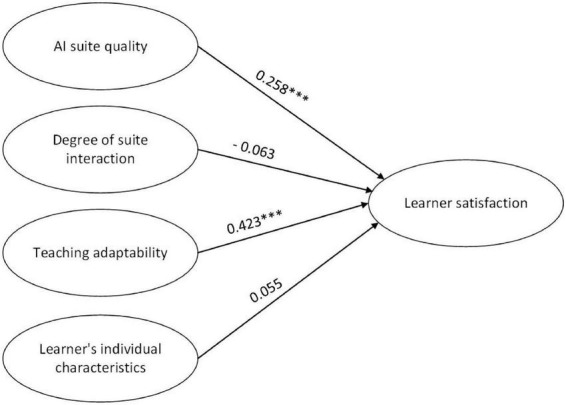
Structural model.

Based on the non-standardized coefficients, both suite quality and improvement in individual students’ abilities significantly influence learner satisfaction. Furthermore, the path coefficient of improvement in individual students’ abilities is high at 0.423, while that of suite quality is only 0.344. It is worth mentioning that appropriateness and interactivity do not significantly impact learner satisfaction. Therefore, learner satisfaction (Y) = 0.258; suite quality (X1) + 0.423; and improvement in individual students’ abilities (X2) + 0.084. Hence, H1 and H3 are accepted, but H2 and H4 are rejected. Compared with suite quality, improvement in individual students’ abilities is more strongly linked to learner satisfaction as shown in Figure 2.

## Discussion and conclusion

### Research discussion

Based on the research results, this study proposes prioritizing suite quality, focusing on improving human–computer interaction, adopting a student-centered approach, and improving the curriculum to enhance the overall effectiveness of the application of the AI science popularization suite in education. Based on the application analysis, the following suggestions regarding curricular integration of the AI science popularization suite are offered.

First, previous studies have shown that college students’ educational level, grades, and understanding of AI significantly influence their ethical cognition of AI in education (Hu et al., 2022). However, the present study finds that the Pearson’s correlation coefficient representing the relationship between undergraduate and graduate students’ grades and learner satisfaction is greater than 0 and that the Sig value exceeds 0.05, which indicates that there is no significant correlation between grades and learner satisfaction. Furthermore, the Pearson’s correlation coefficient between learners’ frequency of exposure to AI suite courses and student satisfaction is greater than 0, with a *P*-value of 0.524 (*P* > 0.05), indicating no correlation here either. The reason may be that this study’s research object is satisfaction with the AI suite, not ethical cognition of AI, and there is no significant correlation between those.

Second, the deep integration of AI technology in classroom courses promotes the development of educational concepts and systems as well as teaching models (Zhao, 2020) and enhances students’ learning satisfaction. While verifying H1, the Pearson’s correlation coefficient of AI suite quality and learner satisfaction was found to be 0.692, and the influence path coefficient was found to be 0.258. Therefore, this study predicts that using the AI suite in classrooms will positively impact learner satisfaction. Regarding the current state of affairs, first, the AI suite is of poor quality and is damaged easily, which will increase the psychological burden on learners using the suite (Hu et al., 2022). Second, the quality problem with the AI suite hinders course task completion, which frustrates learners and greatly lowers students’ learning satisfaction (Liu et al., 2021). Finally, the lack of production standards for the AI suite may, due to environmental influence, result in an unpleasant odor and counterintuitive touch, which could elicit emotion-based rejection among learners.

Third, course interaction data can improve learners’ affinity to a certain extent. While verifying H2, the Pearson’s correlation coefficient of human–computer interaction and learner satisfaction was found to be 0.787, and the influence path coefficient was found to be −0.063, which indicates that there is no obvious negative correlation between the AI suite’s human–computer interaction and learner satisfaction. This result suggests that the human–computer interaction aspect of the AI science suite should be enhanced. The interaction between “AI and pedagogy” is an important standard and yardstick for the development and application of AI (Pedro et al., 2019). First, man–machine error reporting is crucial to allow students to accurately understand how the AI suite operates and to promote the deep integration of AI and subject teaching (Yin et al., 2018). Second, while using the kit, students should avoid spending an excessive amount of time attempting to solve any given problem, as this will lower learner satisfaction. Finally, the following are recommended: increase the degree of interactivity between the suite and learners; avoid unilateral AI output and unilateral student input; and give students’ creativity free rein (Yin et al., 2018).

Fourth, integrating AI into teaching, revolutionizing the conventional teaching mode of theoretical courses, and implementing incremental practical training at different levels can contribute greatly to the cultivation of students’ engineering and innovation abilities (Zhao, 2020). While verifying H3, the Pearson’s correlation coefficient of teaching adaptability and learner satisfaction was found to be 0.778, and the influence path coefficient was found to be 0.423, which indicates that teaching adaptability is significantly positively correlated with learner satisfaction. Therefore, this study asserts that the AI science suite can be used as a teaching aid. To apply the suite in classrooms, first, the suite’s degree of difficulty should be adjusted to suit learners’ characteristics. Specifically, dynamic changes in students’ emotional state should be monitored, as should learners’ growth trajectory. Doing so will provide the information necessary to apply the AI suite at an appropriate level of difficulty for learners in different grades (Wu et al., 2021). Second, the AI suite should be compatible with the course’s teaching objectives, so that it may contribute to fulfilling them. Finally, the AI suite should be applied in practice in the context of maker education to help learners understand key points and find solutions to the challenges that arise in the course, while also improving students’ practical abilities, in addition to making learning fun.

Fifth, satisfaction with the AI suite depends on the degree of match between expectations of the suite’s educational performance and the actual perceived service level (Liu et al., 2006). Individuals’ expectations differ, resulting in varying satisfaction levels. While verifying H4, the Pearson’s correlation coefficient representing the relationship between improvement in the individual students’ abilities and learner satisfaction was found to be 0.876, and the influence path coefficient was found to be 0.055, which indicates that there is no significant positive correlation between individual learner characteristics and learner satisfaction. Therefore, this study recommends the adoption of a student-centered approach when using the AI suite to improve students’ logical thinking and hands-on abilities as well as creativity and promote students’ all-round development. Application of the AI suite should involve the monitoring of improvements in students’ abilities and incremental adjustment of the suite’s difficulty level to avoid using a setting that is either below or above the course’s difficulty level.

### Practical and theoretical implications

At first, Luhmann et al. (2017) pointed out that technical fear of AI manifests in sociological cognition and reflective cognitive methods associated with risk problems. This research method can improve AI science suite application quality in maker education, discourage learners’ negative mindset regarding using the AI science suite to complete maker education classroom tasks and improve students’ satisfaction with the AI suite.

Second, this study helps to avoid unilateral AI output or learner input in maker education (Wang, 2019). Enhancing the degree of interaction between AI and students can give students’ creativity free rein and improve students’ comprehensive and logical thinking abilities.

Finally, this study will help schools and teachers prioritize kit quality, focus on improving human–computer interaction, adhere to the student-centered approach Rogers (1978) proposed in 1952 (Liu, 2012), and aim to improve curriculum suitability by implementing the application analysis-derived suggestions regarding usage of the AI science popularization kit into the practical execution of the maker education curriculum to fully reflect Dewey’s idea of “learning by doing” in maker pedagogy within the larger framework of democracy and education (Dewey, 1974; Chen, 2013).

In general, the application of the AI science popularization suite to the maker teaching process will affect learner satisfaction. It is necessary to simultaneously consider suit quality, the degree of interactivity, teaching adaptability, and learners’ individual characteristics, especially to maintain overall teaching adaptability. Ignoring any of these factors will lower students’ satisfaction with the maker education.

### Limitations and future research

The present study has some limitations, and there remain problems to be solved in the future.

First, the sample does not include all the universities in China, and the degree of penetration of the maker movement differs across provinces. In a similar vein, the effect of regional variance in AI suite characteristics on learner satisfaction with maker education is worth studying.

Second, the sample is limited to specific genders and grades. Future research should consider expanding the sample to avoid sampling-related limitations.

Finally, based on a review of the existing research on factors affecting learner satisfaction with the use of the AI science popularization suite in maker education, it is important to specifically explore suite quality, curriculum suitability, improvement in individual students’ abilities, human–computer interaction, etc.

In the future, we should further improve learners’ satisfaction with using the AI suite in maker education, improve the effectiveness of maker teaching, and promote the deepening of the integration of the AI suite in maker education.

## Data availability statement

The original contributions presented in this study are included in the article/supplementary material, further inquiries can be directed to the corresponding author.

## Author contributions

YFL: formal analysis, investigation, writing – original draft, and writing – review and editing. ZJ: methodology, investigation, visualization, and writing – review and editing. YXL: conceptualization and writing – original draft. JH: writing – original draft and writing – review and editing. All authors contributed to the article and approved the submitted version.

## Conflict of interest

The authors declare that the research was conducted in the absence of any commercial or financial relationships that could be construed as a potential conflict of interest.

## Publisher’s note

All claims expressed in this article are solely those of the authors and do not necessarily represent those of their affiliated organizations, or those of the publisher, the editors and the reviewers. Any product that may be evaluated in this article, or claim that may be made by its manufacturer, is not guaranteed or endorsed by the publisher.
